# Random Forest in Clinical Metabolomics for Phenotypic Discrimination and Biomarker Selection

**DOI:** 10.1155/2013/298183

**Published:** 2013-02-02

**Authors:** Tianlu Chen, Yu Cao, Yinan Zhang, Jiajian Liu, Yuqian Bao, Congrong Wang, Weiping Jia, Aihua Zhao

**Affiliations:** Center for Translational Medicine and Shanghai Key Laboratory of Diabetes Mellitus, Department of Endocrinology and Metabolism, Shanghai Jiao Tong University Affiliated Sixth People's Hospital, Shanghai 200233, China

## Abstract

Metabolomic data analysis becomes increasingly challenging when dealing with clinical samples with diverse demographic and genetic backgrounds and various pathological conditions or treatments. Although many classification tools, such as projection to latent structures (PLS), support vector machine (SVM), linear discriminant analysis (LDA), and random forest (RF), have been successfully used in metabolomics, their performance including strengths and limitations in clinical data analysis has not been clear to researchers due to the lack of systematic evaluation of these tools. In this paper we comparatively evaluated the four classifiers, PLS, SVM, LDA, and RF, in the analysis of clinical metabolomic data derived from gas chromatography mass spectrometry platform of healthy subjects and patients diagnosed with colorectal cancer, where cross-validation, *R* ^2^/*Q* ^2^ plot, receiver operating characteristic curve, variable reduction, and Pearson correlation were performed. RF outperforms the other three classifiers in the given clinical data sets, highlighting its comparative advantages as a suitable classification and biomarker selection tool for clinical metabolomic data analysis.

## 1. Introduction

Metabolomics [1] or metabonomics [2, 3] is an emerging-omics approach using nuclear magnetic resonance (NMR) spectroscopy or gas chromatography/liquid chromatography-mass spectrometry (GC-MS or LC-MS) technologies. It constitutes a field of science that deals with the measurement of metabolite variations in a biological compartment for the study of the physiological processes in response to xenobiotic interventions that is complementary to organ-specific biochemical and histological findings. Through the analysis of one or several kinds of biofluids including serum, urine, saliva, and tissue samples, the global and dynamic alterations in metabolism can be deciphered [4]. Therefore, metabolomics has been increasingly used in many applications such as identifying metabolite markers for clinical diagnosis and prognosis [5], monitoring the chemical-induced toxicity [6], exploring the potential mechanism of diverse diseases [7], and assessing therapeutic effects of treatment modalities [8, 9]. Univariate and/or multivariate statistical methods are routinely used in metabolomics studies, aiming at successful classification of samples with metabolic phenotypic variations and identification of potential biomarkers while minimizing the technical variations. 

To date, the most widely used classification methods in metabolomic data processing include principal component analysis (PCA), projection to latent structures (PLS) analysis, support vector machine (SVM), Linear discriminant analysis (LDA), and univariate statistical analysis such as Student's *t*-test and analysis of variance (ANOVA) test [10, 11]. We recently applied some of these methods in combination to identify metabolite-based biomarkers in hepatocellular carcinoma [5], gastric cardia cancer [12], knee osteoarthritis [13], oral cancer [14], and schizophrenia [7]. Nevertheless, more effective and robust bioinformatics tools are in critical need for metabolomic data analysis especially when dealing with clinical samples with large individual variability due to diverse demographic and genetic background of patients and various pathological conditions or treatments. 

A machine learning method, random forest (RF), is reported as an excellent classifier with the following advantages: simple theory, fast speed, stable and insensitive to noise, little or no overfitting, and automatic compensation mechanism on biased sample numbers of groups [15]. RF has been widely used in microarray [16–18] and single nucleotide polymorphism (SNP) [19] data analysis achieving good performance. However, in the field of clinical metabolomic data analysis, it has not got enough attention and concern. In addition, no comprehensive performance evaluation about this classifier is reported.

In this research, RF was used in the analysis of a GC-MS derived clinical metabolomic dataset. Its classification and biomarker selection performances were compared with PLS, LDA, and SVM comprehensively. The score plot based on cross validation was used for classification accuracy evaluation. The cross-validation and ROC (receiver operating characteristic) curve were carried out to test their prediction ability and stability. The *R* ^2^/*Q* ^2^ plot was adopted for overfitting measurement. Variable number dependence of the 4 classifiers was explored by eliminating variables step by step. Besides these classification performances, the variable ranking and putative biomarker selection power of RF was examined as well by Pearson correlation.

## 2. Methods

### 2.1. Metabolomic Data Set

Colorectal cancer (CRC) is one of the common types of cancer and the leading causes of cancer death in the world [20]. Urinary samples of 67 CRC patients (67 preoperation samples and 63 matched postoperation ones) and 65 healthy volunteers were collected from the Cancer Hospital affiliated to Fudan University (Shanghai, China). Patients enrolled in this study were not on any medication before preoperative sample collection. The postoperative samples were collected on the 7 day after surgery. Sample collection protocol was approved by the Cancer Hospital Institutional Review Board and written consents were signed by all participants prior to the study. The healthy volunteers were selected by a routine physical examination, and any subjects with inflammatory conditions or gastrointestinal tract disorders were excluded. Other background information such as diet and alcohol consumption was considered during sample selection to minimize the diet-induced metabolic variations. All the samples were collected in the morning before breakfast, immediately centrifuged, and stored at −80°C until analysis. Clinical characteristics of all the samples in this study are provided in Table 1. All the samples were chemically derivatized and subsequently analyzed by GC-MS following our previously published procedures [21].

The acquired MS data were pretreated and processed according to our previously published protocols [5, 7]. A total of 187 variables (areas of peaks, denoting concentrations of metabolites), 35 metabolites were obtained from the spectral data analysis. Normalization (to the total intensity to compensate for the overall variability during sample extraction, injection, detection, and disparity of urine volume), mean centering, and unit variance scaling of the data sets were performed prior to statistical analysis. Finally, the data set contains 187 variables and 195 samples. Two cases: (a) Normal versus CRC patients (preoperative) and (b) Preoperative versus postoperative patients were involved for analysis.

### 2.2. Random Forest

Random forest (RF), developed by Breiman [22], is a combination of tree-structured predictors (decision trees). Each tree is constructed via a tree classification algorithm and casts a unit vote for the most popular class based on a bootstrap sampling (random sampling with replacement) of the data. The simplest random forest with random features is formed by selecting randomly, at each node, a small group of input variables to split on. The size of the group is fixed throughout the process of growing the forest. Each tree is grown by using the CART (classification and regression tree) methodology without pruning. The tree number of the forest in this study is set to be 200, the number of input variables tried for each node is the square root of the number of total variables, and the minimum size of the terminal nodes is set to be 2. The “score” of RF is the scaled sum of votes derived from the trained trees for out-of-bag samples.

RF includes two methods for measuring the importance of a variable or how much it contributes to predictive accuracy. The default method is the Gini score (the method of this study). For any variable, the measure is the increase in prediction error if the values of that variable are permuted across the out-of-bag observations. This measure is computed for every tree, then averaged over the entire ensemble, and divided by the standard deviation over the entire ensemble. Therefore, the larger the Gini score is (ranges from 1 to 100), the more important a variable is.

Please refer to the appendices for the introduction of other classifiers (PLS, SVM, and LDA).

### 2.3. Evaluation of Classification Performance

The classification performance of RF as well as PLS, LDA, and SVM can be evaluated and compared using several approaches: cross-validation, *R* ^2^/*Q* ^2^ plot, ROC, and reduction of variable number.

#### 2.3.1. Cross-Validation: Prediction Ability and Stability

Two types of cross-validations: *k*-fold and *x*% hold out were employed to estimate the prediction ability with low bias and low variance. (1) In the *k*-fold cross-validation, the training set was first divided into *k* subsets (the folds) of approximately equal size. Sequentially each subset was tested using the classifier trained on the remaining *k*−1 subsets, where *k* was set to be 7 and 10 in this study. (2) Holdout cross-validation is similar to *k*-fold cross-validation except for the repeatedly (100 times) random selection of the two mutually exclusive training and testing (holdout) subsets in accordance with a given ratio. This method was used with an understanding that the more instances left for the holdout set, the higher the bias of the estimate would be. On the other hand, fewer holdout set instances mean that the confidence interval for the accuracy would be wider. Besides accuracy and prediction ability, the repeated holdout cross-validation was used to test the stability of a classifier. The holdout ratios were set as 10%, 15%, and 33%, respectively, on all the classifiers.

#### 2.3.2. R^2^/Q^2^ Plot—Overfitting

Consider

(1) $$\begin{array}{c} R^{2}=1-\frac{\sum_{i=1}^{n}{\left(y_{i}-{\hat{y}}_{i}\right)}^{2}}{\sum_{i=1}^{n}{\left(y_{i}-{\overset{-}{y}}_{i}\right)}^{2}},\\ Q^{2}=1-\frac{\sum_{i=1}^{n}{\left(y_{i}-{\hat{y}\;}_{\left(i\right)}\right)}^{2}}{\sum_{i=1}^{n}{\left(y_{i}-{\overset{-}{y}}_{\left(i\right)}\right)}^{2}}. \end{array}$$

In the equations, *n* represents total number of samples, *y*_*i*_ is the predicted class (0 or 1) of the *i*th sample when all the samples are used for model building, ${\hat{y}}_{i}$ is the actual class, ${\overset{-}{y}}_{i}$ is the average of the predicted class, and *y*_(*i*)_ is the predicted class when all the samples except the *i*th sample are used for model building (leave one out cross-validation).

The criteria for classifier validity are as follows. (1) All the *R* ^2^ and *Q* ^2^ values on the permuted data set are lower than those on the actual data set. (2) The regression line of *Q* ^2^ (line joining the actual *Q* ^2^ point to the centroid of the cluster of permuted *Q* ^2^ values) has a negative intercept value on the vertical axis.

#### 2.3.3. Receiver Operating Characteristic (ROC): Diagnosis Potential

ROC analysis is a classic method from signal detection theory and is now commonly used in clinical research [23]. ROC of a classifier shows its performance as a tradeoff between specificity and sensitivity. Sensitivity is defined as the proportion of subjects with disease whose tests is positive, and calculated by the formula, TruePositive/(TruePositive+FalseNegative). Specificity, on the other hand, is defined as the proportion of subjects without disease whose tests is negative, and calculated in the formula, TrueNegative/(TrueNegative+FalsePositive). Typically, 1-specificity is plotted on the *x*-axis and sensitivity is plotted on the vertical axis. All the predictive behavior of a classifier can be represented by the points in the ROC curve independent of class distributions or error cost [23]. The area under the ROC curve (AUC) is a statistic summary of its diagnostic potential. 

#### 2.3.4. Variable Number Dependence

Generally, too many irrelevant variables are liable to result in overfitting decisions, whereas differences between groups cannot be extracted and depicted completely if crucial variables are not concerned [24]. Variable number dependence is therefore a necessary factor for classifier performance evaluation. 

To avoid bias, it is advisable to rank and eliminate variables one by one. Initially, the whole dataset is taken when a classifier is computed. Then, a list of variables in descending order relative to classification importance is established and the variable in the end is eliminated for subsequent analysis. This process is repeated until only one variable is left for classifier building. The last few variables are of great potential to be biomarkers for separating the groups.

### 2.4. Evaluation of Biomarker Selection Performance

Prediction ability and stability, overfitting, diagnosis potential, and variable number dependence are important aspects for a classifier. Variable ranking and biomarker selection is of the same importance in metabolomics study.

For RF, variables are ranked by Gini score, a measurement of average accuracy of all trees containing a particular variable [22]. For PLS, the conventional VIP (variable importance in projection) value is used for variable ranking. For LDA, the coefficients of variables in the discriminant function indicate their importance. As to SVM, the importance of variables is evaluated by the SVM recursive feature elimination (SVM-RFE) algorithm [25].

As each classifier possesses its own algorithm for variable importance ranking with its own strength and weakness, the Pearson correlation coefficient of every two ranks was used to evaluate their consistency and the rank of *t*-test (by ascending order of variable *P* values) was taken as an unbiased reference. The consistency comparison was conducted on two levels: ranks of all variables and ranks of identified metabolites. 

All the metabolites were identified and verified by public libraries such as HMDB, KEGG, and/or reference standards available in our laboratory. 

All the classifiers andevaluation methods were carried out using Matlab toolbox (Version 2009a, Mathworks).

## 3. Results and Discussion

### 3.1. Classification Performance

**Figure 1:**
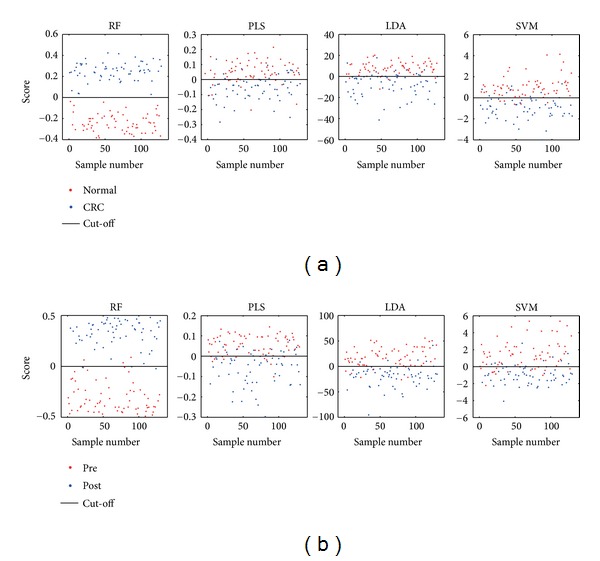
Classification score plots of RF, PLS, LDA, and SVM on cases (a) and (b) based on urinary metabolomic data derived from GC-MS. *x*-axis is the sample index and *y*-axis is the classification scores. (a) Normal versus CRC, (b) pre versus post.

RF as well as PLS, LDA, and SVM were applied on the dataset for the two comparative cases (Figures 1(a) and 1(b)). In Figure 1(a), red and blue dots represent the normal and CRC patients, respectively. *x*-axis is the sample index and *y*-axis is the corresponding “score” of every classifier. RF achieved the best separation with no miss-classified samples (the accuracy is 100%). The performance of SVM, LDA, and PLS was good, with descending accuracy of 90.9%, 87.1%, and 82.6%, respectively. Similarly, Figure 1(b) shows the separation between CRC preoperative patients (red dots) and CRC postoperative patients (blue dots). RF yielded 95.4%, a higher classification accuracy than that of LDA, SVM, and PLS, which achieved 90.8%, 80.8%, and 80.8%, respectively.

### 3.2. Prediction Ability, Stability, Overfitting, and Diagnostic Ability Evaluation

The accuracy of classification is crucial for a classifier, while other classification behaviors such as prediction ability, stability, degree of overfitting and diagnostic ability are of equal significance as well.

**Figure 2:**
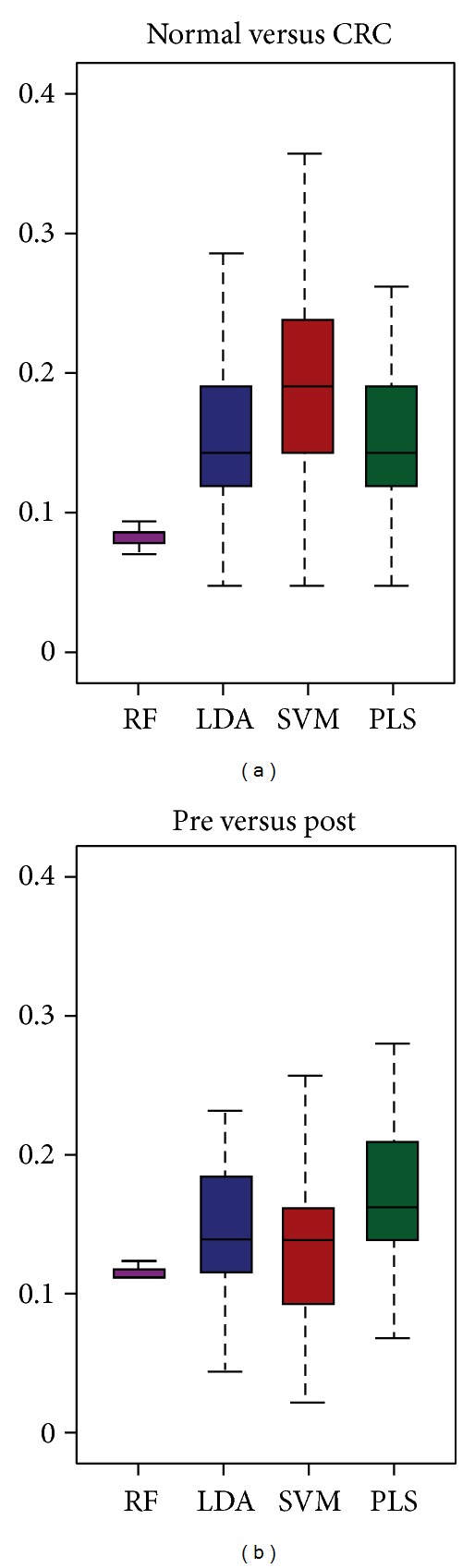
Box plots of holdout cross-validation error rates (*y*-axis) on randomly selected 33% samples (repeated 100 times) on (a) normal versus CRC, and (b) pre versus post. Purple: RF, blue: PLS, brown: LDA, and green: SVM.

The holdout cross-validation results (33% holdout samples, 100 times) of RF (purple), PLS (blue), LDA (red), and SVM (green) on the two cases are presented as box plots (Figure 2). The *y*-axis denotes the error rate (the smaller, the better). The purple box of RF is always the lowest and shortest in both cases. As to the other three classifiers, their performances are similar showing no significant difference. These results were validated further by more cross-validation results listed in Table 2. As expected, the average error rates and standard deviations (S.D.s) of (1) holdout cross-validations on 10% and 15% samples; and (2) 7-and 10-fold cross-validations by PLS, SVM, and LDA are at almost the same level and are all greater than that of RF. Therefore, RF is the one with highest prediction ability and best stability among all the classifiers.

**Figure 3:**
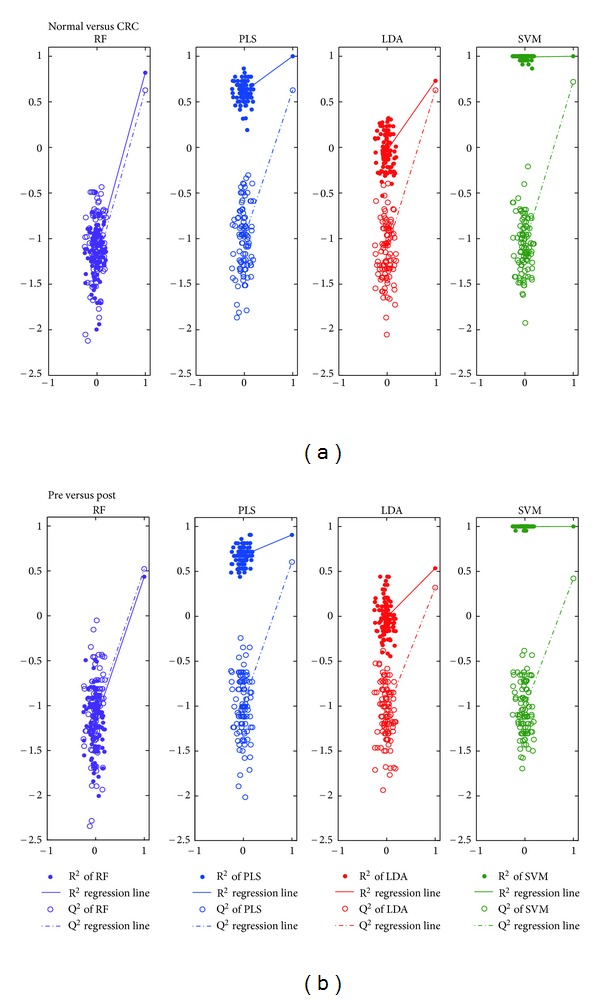
*R* ^2^/*Q* ^2^ plots of 4 classifiers on 2 cases. Correlation between the actual *y*-variable and the permuted *y*-variable (*x*-axis) versus the *R* ^2^ and *Q* ^2^ values (*y*-axis) on (a) Normal versus CRC, and (b) pre versus post. Dot: *R* ^2^, circle: *Q* ^2^, straight line: *R* ^2^ regression line, dash dot line: *Q* ^2^ regression line. purple: RF, blue: PLS, brown: LDA, and green: SVM.

Figures 3(a) and 3(b) display the correlation between the actual *y*-variable and the permuted *y*-variable (*x*-axis) versus the *R* ^2^/*Q* ^2^ values (*y*-axis) of RF (purple), PLS (blue), LDA (red), and SVM (green) on the two cases, respectively. A dot denotes *R* ^2^ and a circle represents *Q* ^2^. The straight line and dash dot line are the regression lines (lines linking the value from the actual data set and the 100 permuted ones) of *R* ^2^ and *Q* ^2^, respectively. Obviously, the *R* ^2^/*Q* ^2^ values of RF on the permuted data sets are all under zero and are well lower than those on the actual data set. Consequently, RF does not over fit the data and so will give out reliable result on new samples. As to SVM, its *R* ^2^ values on the permuted data sets are slightly lower than or close to that on actual data set, although most of its *Q* ^2^ values on the permuted data sets are under zero and lower than those on the actual data set. Hence, SVM is overfitted and false positive result is prone to appear. This probably caused by its dependence on kernel functions and support vectors.

**Figure 4:**
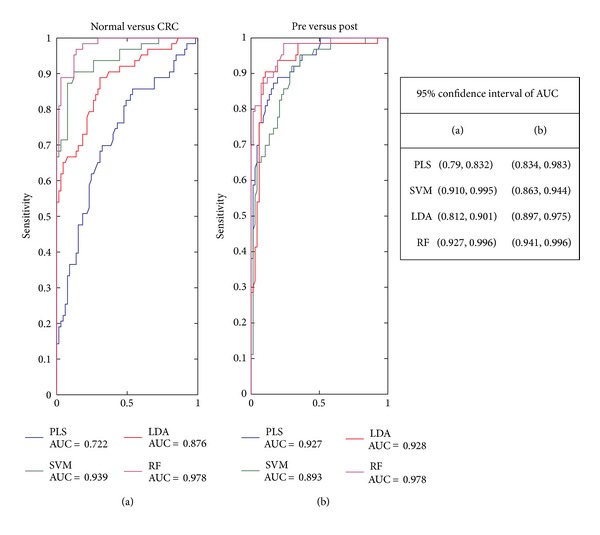
Receiver operating characteristic curves of 4 classifiers on 2 cases. Receiver operating characteristic curve and area under curve (AUC) of PLS (blue), LDA (brown), SVM (green), and RF (Purple) on (a) Normal versus CRC and (b) pre versus post.

The ROC curve coupled with its area under the curve (AUC) is a common method used to estimate the diagnosis potential of a classifier in clinical applications. A larger AUC indicates higher prediction ability. The ROC curves and AUC values of all the classifiers in the two cases are plotted in Figure 4. RF outperforms the others once more with the greatest AUC values (AUC > 0.97).

### 3.3. Variable Number Dependence

**Figure 5:**
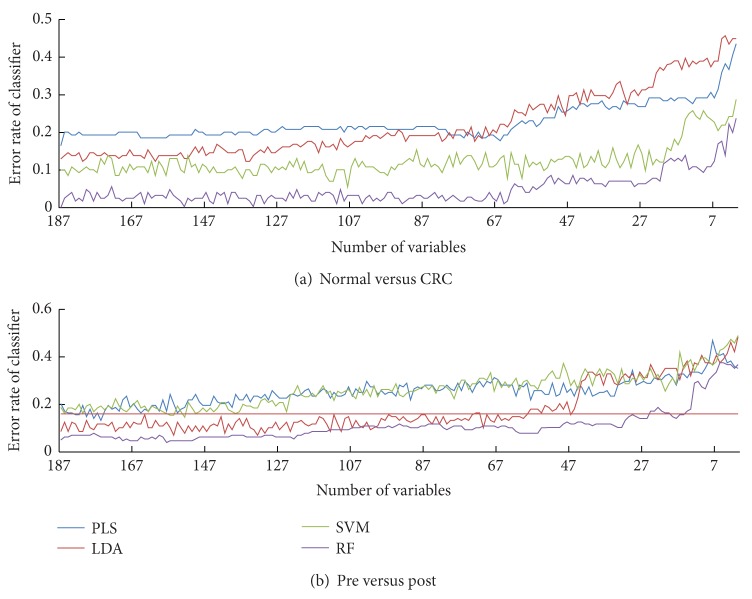
Variable dependence plots of 4 classifiers on 2 cases. Error rate (*y*-axis) of RF (Purple), PLS (blue), LDA (brown), and SVM (green) with decreasing variable number (*x*-axis) in (a) Normal versus CRC, and (b) pre versus post.

Figures 5(a) and 5(b) show the classification error rates (*y*-axis) against the number of variables (*x*-axis) involved in the two cases, respectively. It can be seen that with the decrease of variable number used for classifier building, all the curves keep stable initially, and then rise gradually. Further reduction of variables degrades the classifier performance heavily because of the shortage of useful information. The point (or short section) where the curve begins to rise correlates to the optimum number of variables for classifier building. Additionally, RF usually needs fewer variables to achieve the same error rate as the other three classifiers. In case (B), for example, when the error rate is restricted to be less than 0.18 (the red line), RF needs minimum 10 variables whereas PLS, LDA, and SVM require about 150, 45, and 125 ones at least.

### 3.4. Putative Biomarker Selection

**Figure 6:**
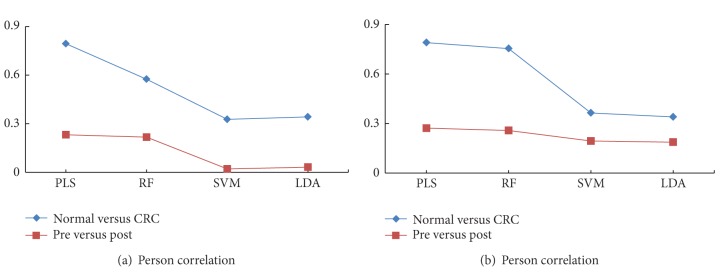
Pearson correlation values between ranks of *t*-test and each classifier in 2 cases by (a) all variables and (b) identified metabolites. *x*-axis is the classifiers and *y*-axis is the Pearson correlation coefficients of classifiers and *t*- test. Blue line: case (A) (Normal versus CRC) and brown line: case (B) (pre versus post).

Variable number dependence section is to evaluate whether and how much the performance of RF depends on the number of variables involved. This section is to evaluate its capability on important variable (putative biomarker) selection. The Pearson correlation matrixes of ranks from every two classifiers (including *t*-Test) based on all variables (A-B) and identified metabolites (C-D) in the two cases are listed in Table 3. On the whole, RF, PLS and *t*-Test have good consistency with each other (high Pearson correlation coefficients) regardless of whether all variables (Figure 6(a)) or identified metabolites (Figure 6(b)) are involved.

**Figure 7:**
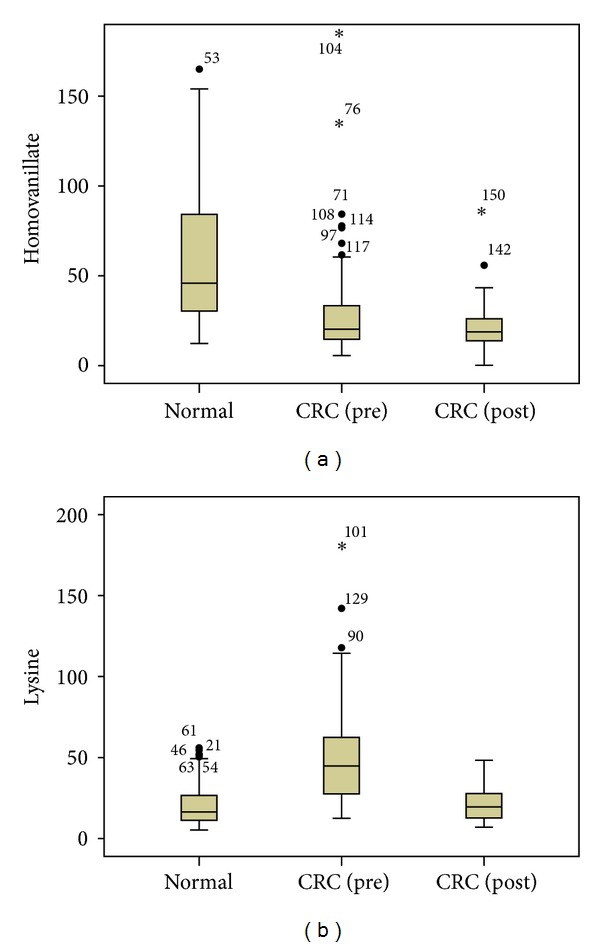
Box plots of significant metabolites selected by RF (case A) only. *x*-axis is the groups (normal, CRC (pre), and post) and *y*-axis is the intensity of metabolites ((a) for homovanillate and (b) for lysine).

Interestingly, in Table 3, the highest and second highest correlation coefficients are 0.794 for PLS and *t*-Test (case A) and 0.756 for RF and PLS (case B) indicating the consistency and mutual complementarity of classifiers. All the important metabolites selected by both *t*-Test (*P* < 0.05) and PLS (VIP > 1) could be filtered by RF (Gini > 50) as well. Consistent with previous metabolomics study, dysregulated metabolic pathways, such as glycolysis, TCA cycle, urea cycle, pyrimidine metabolism, polyamine metabolism as well as gut microbial-host cometabolism were observed [26]. Additionally, more significant metabolites in the above pathways could be found by RF (case A) providing complementary information for CRC study.  Figure 7 presents some box plots of intensity in corresponding groups.

## 4. Conclusion

In this study, RF was applied successfully in metabolomic data analysis for clinical phenotype discrimination and biomarker selection. Its various performances were evaluated and compared with the other three classifiers PLS, SVM, and LDA by two types of cross-validations, *R* ^2^/*Q* ^2^ plot, ROC curve, variable elimination, and Pearson correlation. RF demonstrated the best overall performance including accuracy, prediction ability, overfitting, diagnosis potential, stability, and putative biomarker selection, highlighting its potential as an excellent classification and biomarker selection tool for clinical metabolomic data analysis. PLS outperforms the others in variable ranking and putative biomarker selection whereas its classification ability is not satisfactory enough. LDA demonstrates reasonably good performance in classification but its biomarker selection ability is open to question. SVM may be slightly overfitting regardless of its good classification accuracy and diagnosis potential. 

The combinational usage of multiple methods, RF, *t*-Test, and PLS, for example, may provide more comprehensive information for a “global” understanding of the metabolomics or other “omics” data.

## Figures and Tables

**Table 1:** Sample information.

Data set	CRC		
Sample type	urine		
Group	Normal	CRC (preoperation)	postoperation
Number	65	67	63
Age (Mean (minimum, maximum))	55 (38, 74)	59 (40, 76)	60 (40, 77)
Gender (male : female)	23 : 40	35 : 28	36 : 24
Dimension (Sample × variable)	Case A (Normal versus CRC): 132 × 187		
	Case B (Pre versus Post): 130 × 187		

**Table 2:** Averaged error rates and their standard deviations of RF, PLS, LDA, and SVM on 2 cases by 7- and 10-fold cross-validation as well as 10% and 15% hold out cross-validation (100 times).

Case	Evaluation item	RF error rate		PLS error rate		SVM error rate		LDA error rate	
		mean (S.D.)		mean (S.D.)		mean (S.D.)		mean (S.D.)	
(A) Normal versus CRC	7-fold CV	0.071	(8.364*E* − 03)	0.134	(7.845*E* − 02)	0.148	(7.998*E* − 02)	0.227	(9.897*E* − 02)
	10-fold CV	0.069	(7.482*E* − 03)	0.094	(8.990*E* − 02)	0.126	(6.673*E* − 02)	0.188	(1.375*E* − 01)
	15% holdout CV	0.065	(6.545*E* − 03)	0.132	(7.009*E* − 02)	0.117	(7.344*E* − 02)	0.189	(8.206*E* − 02)
	10% holdout CV	0.065	(5.445*E* − 03)	0.121	(8.407*E* − 02)	0.113	(8.572*E* − 02)	0.181	(1.073*E* − 01)

(B) Pre versus post	7-fold CV	0.102	(3.687*E* − 03)	0.130	(7.321*E* − 02)	0.170	(8.069*E* − 02)	0.108	(5.517*E* − 02)
	10-fold CV	0.096	(3.954*E* − 03)	0.169	(1.242*E* − 01)	0.163	(1.524*E* − 01)	0.096	(1.069*E* − 01)
	15% holdout CV	0.088	(3.592*E* − 03)	0.137	(6.687*E* − 02)	0.186	(8.412*E* − 02)	0.127	(7.958*E* − 02)
	10% holdout CV	0.083	(3.188*E* − 03)	0.145	(9.883*E* − 02)	0.161	(8.644*E* − 02)	0.114	(9.375*E* − 02)

**Table 3:** Pearson correlation coefficient matrixes of rank lists by *t*-test, PLS, SVM, and RF in 2 cases based on all variables (A-B) and identified metabolites (C-D).

Method	*t*Rank^a^	PLSRank^b^	RFRank^c^	SVMRank^d^	LDARank^e^
Pearson correlation coefficient matrix based on all variables					

(A) Normal versus CRC					
*t*Rank	1.000	0.794^f^	0.575	0.327	0.342
PLSRank	0.794	1.000	0.574	0.328	0.342
RFRank	0.575	0.574	1.000	0.210	0.256
SVMRank	0.327	0.328	0.210	1.000	0.167
LDARank	0.342	0.342	0.256	0.167	1.000
(B) Pre versus post					
*t*Rank	1.000	0.232	0.217	0.021	0.032
PLSRank	0.232	1.000	0.652	0.066	0.066
RFRank	0.217	0.652	1.000	0.086	0.057
SVMRank	0.021	0.066	0.086	1.000	0.007
LDARank	0.032	0.066	0.057	0.007	1.000

Pearson correlation coefficient matrix based on identified metabolites					

(C) Normal versus CRC					
*t*Rank	1.000	0.753	0.754	0.364	0.340
PLSRank	0.753	1.000	0.756	0.267	0.340
RFRank	0.754	0.756	1.000	0.495	0.308
SVMRank	0.364	0.267	0.495	1.000	0.190
LDARank	0.340	0.340	0.308	0.190	1.000
(D) Pre versus post					
*t*Rank	1.000	0.272	0.258	0.194	0.187
PLSRank	0.272	1.000	0.733	0.048	0.044
RFRank	0.258	0.733	1.000	0.034	0.041
SVMRank	0.194	0.048	0.034	1.000	0.187
LDARank	0.187	0.044	0.041	0.187	1.000

^a^variable rank by the *P* value of *t*-test.

^b^variable rank by the VIP value of PLS.

^c^variable rank by the Gini value of RF.

^d^variable rank by the SVM-REF.

^e^variable rank by the LDA coefficient.

^f^Pearson correlation coefficient of PLS and *t*-test variable rank lists for differentiating Normal and CRC.

## Appendices

### A. Projection to Latent Structures (PLS)

The basic object of PLS is to find the linear (or polynomial) relationship between the superior variable *Y* (a vector indicating sample groups) and the dataset *X* (variables). The modeling consists of simultaneous projections of both the *X* and *Y* spaces on low dimensional hyper planes. The coordinates of the points (projection of *X* and *Y*) on these hyper planes constitute the elements of the matrix *T* (*X* scores), *U* (*Y* scores), *P* (loadings), and *C* (weights). The analysis has the following objectives:

- To well approximate the *X* and *Y* spaces,
- To maximize the correlation between *X* and *Y*. That is, to maximize the covariance between the sample positions (from different groups) on the hyper planes.

The PLS model accomplishing these objectives can be expressed as

(A.1) $$\begin{array}{c} X=TP^{T}+E,\\ Y=UC^{T}+F. \end{array}$$

The model will iteratively compute one component at a time, that is: one vector derived from *X*-scores *T*, *Y*-scores *U*, weights *C* (or *w*), and loadings *P*. The component extraction process will stop when the predictive accuracy obtained in 7-fold cross-validation (*Q* ^2^ value) begin to descend. All the components are calculated in descending order of importance. The “score” of PLS is the score of the first component which contains most information of *X* dataset.

The formula to calculate VIP (variable importance) can be expressed as

(A.2) $$\begin{array}{c} {\text{VIP}}_{AK}=\sqrt{\sum_{a=1}^{A}\left(w_{ak}^{2}*\left(SSY_{a-1}-SSY_{a}\right)\right)*\frac{K}{\left(SSY_{a}-SSY_{A}\right)}}, \end{array}$$

where *A* is the number of components extracted and *K* is the number of variables in dataset *X*. *w*_*ak*_ is the correlation between *X* and *U*(*Y*) expressed by component *a*. *SSY*_*a*_ is the explanation of *Y* by component *a*. *SSY*_*A*_ is the explanation of *Y* by all the components. The Sum of squares of all VIP*s* is equal to the number of variables in the model hence the average VIP is equal to 1.

The VIP*s* derived from the first component is used for variable ranking. Variables with larger VIP, larger than 1 in particular, are the most relevant for explaining *Y* (classification).

### B. Support Vector Machine (SVM)

The key to the success of SVM is the kernel function which maps the data from the original space into a high dimensional (possibly infinite dimensional) feature space. By constructing a linear boundary in the feature space, the SVM produces nonlinear boundaries in the original space. Given a training sample, a maximum-margin hyper plane splits a given training sample in such a way that the distance from the closest cases (support vectors) to the hyper plane (*W* ^*T*^ *X* + *b* = 0) is maximized where *W* is the weight matrix, *X* is the dataset, and *b* is a constant term. The “score” of SVM is computed by Score = *W* ^*T*^ *X* + *b*.

SVM Recursive Feature elimination (SVM-RFE) is a wrapper approach which uses the norm of the weights *W* to rank the variables (the larger the norm of *W* is, the more important the corresponding variable is). Initially whole of the data is taken and a classifier is computed. The norm of *W* is computed for each of the features and the feature with the smallest norm is eliminated. This process is repeated till all the features are ranked.

Linear kernel was used for SVM classification and feature selection. This kernel was chosen to reduce the computational complexity and eliminate the need for retuning kernel parameters for every new subset of variables. Another important advantage of choosing a linear kernel is that the norm of the weight *W* can be directly used as a ranking criterion; however this is not possible in other kernels such as the radial basis function kernel or a sigmoid kernel.

### C. LDA

LDA adopts a linear combination of variables (*F*_LDA_ = *WX* + *E*) as a predictor to characterize or separate two or more classes of objects or events.

Different with PLS which look for linear combinations of variables to best explain both the data set *X* and the superior variable *Y*, the criterion of LDA is to maximize the ratio (*S*) of the variance between the classes to the variance within the classes:

(C.1) S=σbetween2σwithin2=(W·μy=1−W·μy=0)2W′∑y=1W+W′∑y=0W=(W·(μy=1−μy=0))2W′(∑y=0+∑y=1)W,

where *σ* ^2^ is the variance between or within groups, *μ* is the mean of variables from corresponding group, and *W* is the coefficients of variables.

The “score” of LDA is the *F* value of testing samples and the coefficients *W*are used for variable ranking.

## Abbreviations

GC-MS: Gas chromatography mass spectrometry
RF: Random forest
LDA: Linear discriminant analysis
SVM: Support vector machine
PCA: Principal component analysis
PLS: Projection to latent structures
ROC: Receiver operating characteristic
CRC: Colorectal cancer
NMR: Nuclear magnetic resonance
MS: Mass spectrometry.
